# Editorial: New methods, techniques and applications in clinical glycoproteomics

**DOI:** 10.3389/fmolb.2023.1170818

**Published:** 2023-03-03

**Authors:** Yong Zhang, Wenjuan Zeng, Yang Zhao, Hao Yang

**Affiliations:** ^1^ NHC Key Lab of Transplant Engineering and Immunology, Institutes for Systems Genetics, West China Hospital, Sichuan University, Chengdu, China; ^2^ Mass Spectrometry Engineering Technology Research Center, Center for Advanced Measurement Science, National Institute of Metrology, Beijing, China

**Keywords:** clinical glycoproteomics, glycoprotein, glycosylation, mass spectrometry, PTM

Protein glycosylation is a complex post-translational modification (PTM) and plays a crucial role in various physiopathological processes, particularly in the progression of many diseases (Xu et al., 2022). However, due to the micro- and macro-heterogeneity of glycosylation, novel methods and techniques are required to quantitatively characterize N/O-glycosylation and extend it to clinical applications. In recent years, valuable approaches have emerged for the identification of glycoproteins/glycopeptides/glycosites, characterization of glycan structures, biomarker discovery for disease diagnosis, glycoprotein engineering, and functional analysis of glycoproteins. These include some novel glycopeptide enrichment materials (Zhang et al., 2019; Zhang et al., 2020), mass spectrometry (MS) fragmentation techniques (Ye et al., 2019; Zhang et al., 2021; Luo et al., 2022; Mao et al., 2022), and data analysis software (Lu et al., 2020; Kawahara et al., 2021; Shen et al., 2021).

The aim of the Research Topic “*New methods, techniques and applications in clinical glycoproteomics*” is to provide methods and techniques for clinical glycoproteomics research. This Research Topic contains many high-quality articles that introduce new methods for glycoprotein analysis. Du et al. proposed a new integrative strategy for the tandem enrichment of N-glycopeptides and phosphopeptides using HILIC and TiO_2_ microparticles. This strategy is more efficient and sensitive than separately enriching the two PTM peptides and allows for the simultaneous characterization of glycosylation and phosphorylation statuses in limited clinical samples. In addition to the typical glycopeptide profiling in glycoproteomic studies, it is possible to achieve direct analysis of the proteoform profiles of specific glycoproteins using size-exclusion chromatography coupled to native MS detection (SEC-nMS). Jager et al. employed SEC-nMS to compare the inter- and intra-donor proteoform profiles of Alpha-1-Antitrypsin (A1AT) in serum and milk. They observed differences in the abundance of distinct N-glycoforms of A1AT, including branching and fucosylation, which contributed to inter-donor proteoform variability.

The development of new methods and technologies is expanding the application range of glycoproteomics, including clinical glycoproteomics, single-cell glycoproteomics, quantitative glycoproteromics, structure-specific glycoproteomics, spatial glycoproteomics, top-down glycoproteomics, and integrative glycoproteomics (Chau et al., 2023). As this Research Topic focuses on clinical glycoproteomics, we collected several studies associated with the application of glycoproteomics in the clinical field. Ren et al. summarized the development of MS methods in the area of N-glycoproteomics and N-glycomics and reviewed the technical advances in N-glycosylation analysis for different kidney diseases. Since protein glycosylation is closely involved in the progression of many diseases, it holds great promise as a biomarker for clinical diagnosis and treatment.Zhang et al. observed significant changes in plasma IgA1 O-glycosylation patterns between IgA nephropathy patients and non-IgA nephropathy disease controls, and demonstrated that N-acetylgalactosamine and galactose in plasma IgA1 could be used as a diagnostic biomarker for IgA nephropathy. Protein glycosylation also attracts signification attention as a drug target in cancer therapy, and various glycosylation inhibitors have been developed. In this Research Topic, Cao et al. applied a nascent glycoproteomic method to investigate the effect of N-linked glycosylation inhibitor-1 (NGI-1) in hepatocellular carcinoma. They found that NGI-1 suppressed the expression of glycosylated lysosome-asscociated membrane protein-2, concomitant with the occurrence of lysosomal defects and autophagy, which may mediate the cytotoxicity of NGI-1 in cancer treatment. Besides the influence on *in vivo* proteins, glycosylation also affects the structure and function of proteins expressed *in vitro*, particularly therapeutic monoclonal antibodies (mAbs). Kim et al. used an MS-based glycoproteomic approach to characterize and compare the N- and O-glycopeptides profiles of Infliximab’s biologics and biosimilar. They discovered a novel N-glycosylation site and O-glycopeptide from distinct biopharmaceuticals, indicating that site-specific glycopeptide analysis could be a useful technique to evaluate therapeutic mAbs and glycoprotein products.

In conclusion, the articles collected in this Research Topic may promote the development of clinical glycoproteomics methodologies and expand their applications.
